# Effects of Aromatherapy with Lavender and Peppermint Essential Oils on the Sleep Quality of Cancer Patients: A Randomized Controlled Trial

**DOI:** 10.1155/2020/7480204

**Published:** 2020-03-25

**Authors:** Sahar Hamzeh, Roya Safari-Faramani, Alireza Khatony

**Affiliations:** ^1^Student Research Committee, Kermanshah University of Medical Sciences, Kermanshah, Iran; ^2^Research Center for Environmental Determinant of Health, Kermanshah University of Medical Sciences, Kermanshah, Iran; ^3^Clinical Research Development Center, Imam Reza Hospital, Kermanshah University of Medical Sciences, Kermanshah, Iran

## Abstract

One of the problems of cancer patients is sleep disorder. Given the absence of studies on comparing the effect of inhalation aromatherapy with lavender and peppermint on the sleep quality of the cancer patients, this study was performed to compare the effect of inhalation aromatherapy with lavender and peppermint essential oils on the sleep quality of cancer patients. For this purpose, 120 patients were randomly allocated to three groups of lavender, peppermint, and control. The intervention groups received three drops of the essential oil for 7 days. In the control group, aromatic distilled water was used instead. Pittsburgh Sleep Quality Inventory (PSQI) was used. Before the intervention, no significant difference was observed between the mean PSQI scores of three groups, while the difference was statistically significant after the intervention. The mean PSQI scores were lower in lavender and peppermint groups than in the control group. Aromatherapy can improve the sleep quality of cancer patients. To confirm the findings, more studies should be done.

## 1. Introduction

Cancer changes many aspects of life [1] and causes negative consequences such as sleep disorder [2]. Evidence indicates that one-third of the cancer patients suffer from sleep disorder [3] and do not have acceptable sleep quality [1, 4]. Studies have revealed that 44%–48% of the cancer patients' prescriptions contain sleeping medications and 28%–37% of the patients take these medicines [5]. Cancer patients sometimes have a severe sleep disorder and need medical intervention [6]. One of these interventions is the use of benzodiazepines, which have negative side effects despite their effectiveness, such as dependency and tolerance [7, 8]. Another method is a complementary medicine known as a low-risk, cost-effective, easy, and low-side effect therapeutic method that is developing all around the world, especially in developing countries. One of the branches of this method is aromatherapy [9], in which essential oil is combined with nasal epithelium receptors and a nerve signal is transmitted to the brain, the limbic system, and the thalamus, which leads to release of endorphins and serotonin [10, 11]. Among the plants whose essential oil is used in aromatherapy, one can name lavender and peppermint. Lavender oil (scientific name: *Lavandula angustifolia*; family: *Lamiaceae*) is one of the most harmless essential oils with no toxicity reported so far [12, 13]. The main components of the lavender are linalool and linalyl acetate [14]. These two components have sedative, antinociceptive, and antispasmolytic effects because of stimulating the parasympathetic system [15]. Linalyl acetate also has narcotic and sedative effect [16]. Another medicinal herb used in aromatherapy is peppermint from the Lamiaceae family [17]. Menthol is one of the chemical compounds of peppermint [18]. Menthol affects Kappa Opioid receptors, blocks the transmission of pain signals, and reduces pain [19]. In addition, peppermint affects the hypothalamus by stimulating the olfactory pathways and decreases the corticotropin-releasing hormone, which reduces cortisol secretion from the adrenal gland, thereby reducing anxiety [20]. The results of studies on the effect of lavender essential oil on the sleep quality are contradictory and challenging. The results of some studies have indicated the positive effect of lavender on reducing insomnia and improving sleep quality [21–24]. Some studies, however, have reported no positive effects [25–27]. To the best of our knowledge, a limited number of studies have been done on the effect of peppermint on the sleep quality of cancer patients, which have shown its positive effect on improving the sleep quality [28, 29]. The results of a systematic review performed in 2016 showed the low reliability of most studies in the field of aromatherapy in cancer patients, hence, suggesting further studies in this regard [30].

Given the absence of studies on comparing the effect of inhalation aromatherapy with lavender and peppermint on the sleep quality of the cancer patients, the present study was performed to compare the effect of inhalation aromatherapy with lavender and peppermint essential oils on the sleep quality of cancer patients.

## 2. Materials and Methods

### 2.1. Study Design

The present randomized, controlled, clinical trial was conducted from March to July 2018.

### 2.2. Hypothesis

Inhalation aromatherapy with essential oils of lavender and peppermint can improve the sleep quality of cancer patients.

### 2.3. Sample and Sampling Method

The research population comprised all patients admitted to the oncology ward of Taleghani Hospital in Kermanshah, Iran. According to the results of Afshar et al. study [31], a type I error of 0.05, a type II error of 0.2, and power of 93.3, sample size of 111 were calculated. Considering the probability of exiting of 10% of the study subjects, 120 subjects were recruited through convenience sampling method and 40 subjects were allocated to each of the three groups of lavender, peppermint, and control via random sampling method. The inclusion criteria consisted of consent of the patient's physician, consent of the patient, complete awareness, at least one month of cancer diagnosis, positive olfactory test (according to the patient's statement and nasal examination for lack of obstruction), lack of physical pain, stability of vital sign (blood pressure, pulse, breathing, and temperature), history of no mental illness (according to the patient's statement), no history of using psychiatric drugs (according to the patient's statement), age range 18–65 years, absence of sinusitis and nasal problems, lack of skin allergy, absence of lung cancer, lack of drug, alcohol, or cigarette addiction, absence of acute clinical problems such as nausea and vomiting, no history of respiratory problems such as asthma, no consumption of caffeine an hour before bedtime, score of ≥5 in Pittsburgh Sleep Quality Inventory (PSQI), absence of a primary tumor or metastasis in the central nervous system, and not taking sleeping drugs. The exclusion criteria included the patient's unwillingness to participate in the study, patient transfer to another ward, drug intake or oxygen use during aromatherapy, and the occurrence of any unforeseen incident or crisis.

The first author was responsible for the recruitment of samples. The samples with inclusion criteria were enrolled through a convenience sampling method, who were allocated to intervention and control groups by the second author (statistical consultant). In order to allocate the samples randomly, the formulas were written in Excel software and five-digit random numbers were generated. A number was allocated to a subject and the last number on the right was the name of the group. For example, patients whose last digits were 4, 5, 6, and 7, 8, 9 were allocated to peppermint and control groups, respectively. The sampling was continued until the sample size was completed. The participants were blind to their allocation.

### 2.4. Measurement Instruments

The measurement tools of the present study were a demographic information questionnaire and PSQI. The demographic questionnaire contained items about age, gender, education, marital status, employment status, and diagnosis. PSQI is a self-report standard inventory used to determine the sleep quality. The validity and reliability of the questionnaire have been evaluated and confirmed by previous studies. The internal consistency of the questionnaire was evaluated by Buysse et al., with Cronbach's alpha of 0.83 [32]. The Persian version of PSQI was psychoanalyzed by Moghadam et al., with Cronbach's alpha of 0.77 [33].

PSQI contains nine items, five of which consist of 10 secondary items (a total of 19 items). The questionnaire includes seven components such as subjective sleep quality, sleep latency, sleep duration, habitual sleep efficiency, sleep disturbances, use of sleeping medication, and daytime dysfunction. The items 1–4 are open-ended, short, and one-choice questions and the items 5–9 are multiple choice. The sum of the scores of the seven components yields one global score, which varies between 0 and 21. An earning point equal to or greater than five represents sleep disorder [34, 35].

### 2.5. Intervention

**Figure 1:**
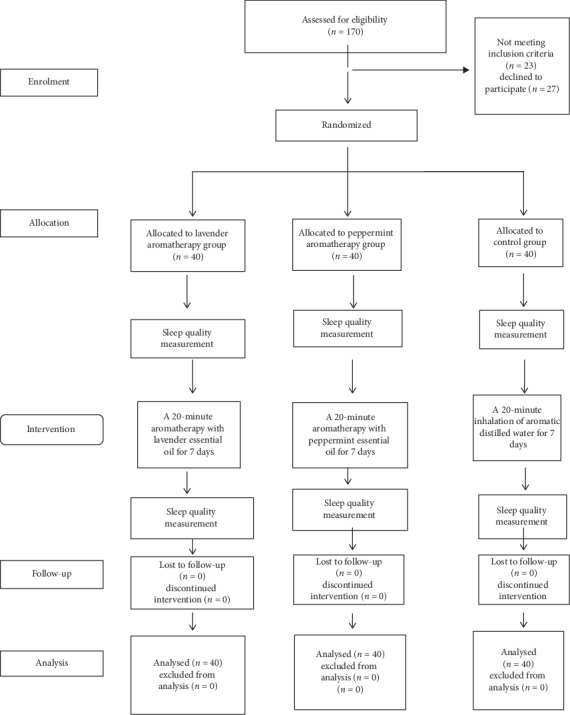
CONSORT diagram of the study.

After obtaining approval from the Ethics Committee of Kermanshah University of Medical Sciences (KUMS), the researcher referred to the oncology ward of Taleghani Hospital affiliated to KUMS and proceeded to select the eligible participants. First, the aims of the study were explained to the patients and their consent to participate in the study was taken. Before the intervention, the demographic form and PSQI were completed by all three groups. The intervention was done by the researcher for 7 days before bedtime at 21 : 00 pm. In peppermint and lavender groups, three drops of peppermint and lavender essential oils were used, respectively. In the control group, for the blinding of the patients, distilled water was mixed with 1% lavender essential oil. The essential oils were produced by Zardband Company (Tehran-Iran) and had 100% purity. Sterile water was produced by Samen Company (Mashhad-Iran). In intervention groups, three drops of the related essential oil were dropped on a cotton ball and attached to the patient collar for 20 min. In the morning of day eight, PSQI was refilled by all three groups (Figure 1). After completing the sample size, the trial stopped.

### 2.6. Data Analysis

The data were analyzed by the Statistical Package for Social Sciences (SPSS v.16.0; SPSS Inc., Chicago, IL, USA) using descriptive statistics (mean and frequency percentage) and inferential statistics. Chi-square test, independent *t*-test, and one-way analysis of variance (ANOVA) were used to ensure the similarity of groups in terms of the distribution of age, gender, marital status, education, occupation, location, and type of cancer, when appropriate. Repeated measure ANOVA applied to assess the effect of aromatherapy and interaction effects of two factors, timing of PSQI (before/after intervention), and the intervention group. The significance level for all tests was less than 0.05.

### 2.7. Ethical Approval

The Ethics Committee of the University approved the study with code: IR.KUMS.REC.1396.647. The study was also registered at the Iranian Registry of Clinical Trials with the registration number: IRCT20100913004736N21. Before starting the study, written informed consent was taken from all the participants. The aims of the study were explained to the participants and their questions were answered. Besides, they were assured their personal information would be kept confidential. In addition, the physician's consent was taken.

## 3. Results

A total of 120 cancer patients participated in this study. Their mean age was 49.47 ± 14.52 years. Most of the subjects were female (*n* = 68, 56.67%), married (*n* = 95, 79.17%), and housewife (*n* = 53, 42.2%), had high-school diploma (*n* = 102, 85%), and suffered from leukemia (*n* = 40, 33.3%). All three groups were almost homogeneous in terms of all demographic variables (Table 1). Since the main inclusion criterion was undesirable sleep quality based on PSQI, all subjects had undesirable sleep quality before recruitment (mean PSQI = 12.62, SD = 2.69). After the intervention, 90% of the subjects (*n* = 36) had undesirable sleep quality and only 10% (*n* = 4) had desirable sleep quality in both lavender and peppermint groups. However, in the control group, 95% (*n* = 38) of the subjects had undesirable sleep quality and only 5% (*n* = 2) had desirable sleep quality.

**Figure 2:**
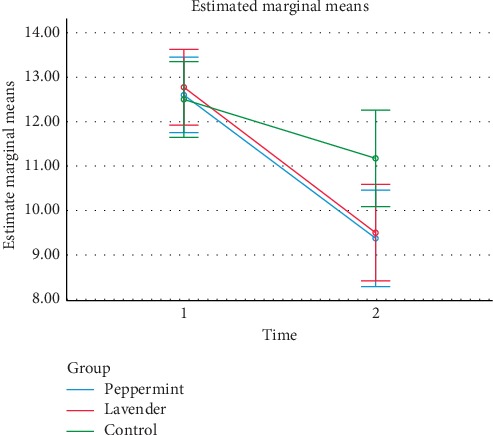
Mean of PSQI before and after intervention in the study groups.

The results revealed no significant difference among study groups in terms of the PSQI mean score before the intervention. The mean score of PSQI in each intervention and control group was improved after the intervention, although this trend was slight in the control group. The result of repeated measure ANOVA showed a nonsignificant effect of group (*F* = 0.989, *p*=0.375). The ANOVA test showed a statistically significant effect for time (*F* = 148.38, *p* < 0.0001) which means that sleep quality in all of the study groups had improved while this trend was slight in the control group. The interaction between time and intervention was statistically significant (*F* = 8.98, *p* < 0.001). The pairwise comparison showed that the mean differences between the peppermint and lavender essential oils groups, as well as the peppermint essential oil and control groups, were −0.15 (*p*=0.817) and −0.85 (*p*=0.190), respectively. Finally, the mean difference between the lavender and control groups was −0.7 (*p*=0.280) (Figure 2 and Table 2).

## 4. Discussion

The aim of the present study was to compare the effect of aromatherapy with lavender and peppermint essential oils on the sleep quality of cancer patients. The results revealed the effectiveness of both essential oils in improving the sleep quality of cancer patients. Regarding the effect of inhalation aromatherapy on sleep quality in cancer patients, there are relatively few studies with main focus on pain, quality of life, vital signs, anxiety, depression, and fatigue in cancer patients. The results of some studies have indicated the positive effect of inhalation aromatherapy on sleep quality. In this regard, Lisa Blackburn et al. investigated the effect of inhalation aromatherapy on insomnia in patients with leukemia. Accordingly, 50 subjects were randomly assigned to one of the aromatherapy and control groups and were studied for two weeks. The aroma used included lavender, peppermint, or chamomile, and patients in the intervention group could select one of them. In the control group, rose water was used. In this study, the subjects were in the aromatherapy group for a week and then they were transferred to the control group in the following week. The results indicated the positive effect of aromatherapy on the sleep quality of cancer patients. Moreover, the most common aromas used in the study were lavender, peppermint, and chamomile, respectively [28]. Our results are in line with this study. However, since the aroma was optional in Lisa Blackburn et al.'s study, it was not possible to say which aroma affected the sleep quality. However, a specific aroma was used in each intervention group in our study. Nevertheless, lavender has a sleep-inducing effect due to its sedative and anxiolytic effects, which makes it relaxing. Following the inhalation of lavender, linalool and linalyl acetate are connected to the olfactory bulb receptors, and their therapeutic effects are applied by the limbic system [28, 36].

However, some studies have reported aromatherapy has no effect on improving sleep quality in cancer patients. In this regard, the effect of inhalation aromatherapy on the quality of life, vital signs, and quality of sleep in 162 patients with breast cancer was investigated in a clinical trial conducted by Tamaki et al. Patients were randomly assigned into one of the two groups, aromatherapy (*n* = 110) and control (*n* = 52). The essential oils used included lavender, orange, and ylang-ylang, of which the patients chose one. Aroma oil was placed at the bedside from 9 : 00 PM the day before surgery to 6 : 00 AM of the surgery day. The results showed no efficacy of aromatherapy in improving the quality of life, vital signs, and sleep quality. According to the Tamaki et al., insufficient sample size can be one of the reasons for finding no subtle changes in the quality of life, vital signs, and sleep quality of breast cancer patients [37]. Our results are not consistent with those of Tamaki et al., due to the use of different types of aromas and the short duration of intervention in the study by Tamaki et al. In the present study, one type of aroma was used for each intervention group and the duration of the intervention was one week. Otaghi et al. investigated the effect of inhalation aromatherapy with lavender on the sleep quality of patients with angiography-candidates using the St. Mary's Hospital Sleep Questionnaire. The results showed that lavender had no effect on sleep quality [27] which was in contrast with the results of our study. The use of different tools for measuring sleep quality and the differences in the nature of cardiovascular and cancerous diseases can be the reasons for this contrast.

In the control group, sleep quality was also significantly improved, although the trend of increasing sleep quality was less than that of the intervention group. Possible causes of sleep quality improvement in the control group may be due to environmental, psychological, and physiological factors [38].

We encountered two limitations in the current study. In our study, the participants were patients with various cancers who used various medicines, which could have affected their response to aromatherapy. Another limitation was that due to the limited number of patients who met the inclusion criteria, they were included regardless of the cancer stage and type, which may influence their response to aromatherapy. The last limitation of the current study was the lack of information about the cancer stage of many patients that could not be considered when analyzing.

## 5. Conclusions

Inhalation aromatherapy with lavender and peppermint essential oils had an identical effect on the sleep quality of the cancer patients. Therefore, this simple and accessible method is suggested to be used to improve the sleep quality of cancer patients. Future studies are suggested to investigate the effects of other aromas as well as other routes of aromatherapy administration, including massage, on the sleep quality of the cancer patients. Further studies are also recommended considering the cancer stage to investigate the effect of aromatherapy on sleep quality.

## Figures and Tables

**Table 1:** Comparison of the demographic variables in the study groups.

Variables	Groups			*p* value
	Peppermint number (%)	Lavender number (%)	Control number (%)	
Age (years)				
≤40	10 (25.0)	13 (32.5)	11 (27.5)	NS^*∗*^
41–60	19 (47.5)	15 (37.5)	17 (42.5)	
≥61	11 (27.5)	12 (30.0)	12 (30.0)	

Sex				
Female	26 (65.0)	24 (35.3)	18 (45.0)	NS
Male	14 (35.0)	16 (60.0)	22 (55.0)	

Marital status				
Single	7 (17.5)	8 (20.0)	10 (25.0)	NS
Married	33 (82.5)	32 (80.0)	30 (75.0)	

Body mass index (kg/m^2^)				
≥18.5	2 (5.0)	0 (0)	3 (7.5)	NS
18.51–24.99	22 (55.0)	21 (52.5)	27 (67.5)	
25–29.99	14 (35.0)	16 (40.0)	10 (25.0)	
≤30	2 (5.0)	3 (7.5)	0 (0)	

Occupation				
Self-employment	11 (27.5)	14 (35.0)	21 (52.5)	NS
Retired	3 (7.5)	4 (10.0)	3 (7.5)	
Housekeeper	21 (52.5)	18 (45.0)	14 (35.0)	
Employee	4 (10.0)	3 (7.5)	1 (2.5)	
Student	1 (2.5)	1 (2.5)	1 (2.5)	

Education				
Nonacademic	34 (85.0)	33 (82.5)	30 (87.5)	NS
Academic	6 (15.0)	7 (17.5)	5 (12.5)	

Diagnosis				
Osteosarcoma	1 (2.5)	2 (5.0)	1 (2.5)	NS
Gastrointestinal cancer	10 (25.0)	6 (15.0)	9 (22.5)	
Lymphoma	1 (2.5)	4 (10.0)	4 (10.0)	
Liver cancer	1 (2.5)	2 (5.0)	2 (5.0)	
Leukemia	11 (27.5)	13 (32.5)	16 (40.0)	
Breast cancer	7 (17.5)	6 (15)	2 (5.0)	
Myeloma	6 (15.0)	5 (12.5)	5 (12.5)	
Ovarian cancer	3 (7.5)	2 (5.0)	1 (2.5)	

Residence				
Rural	32 (80.0)	34 (85.0)	24 (60.0)	NS
Urban	8 (20.0)	6 (15.0)	16 (40.0)	

^*∗*^Nonsignificant.

**Table 2:** Comparing the study groups in terms of sleep quality before and after intervention.

Study groups	Before the study		After the study		*p* value^*∗*^		
	Mean ± SD	95% CI	Mean ± SD	95% CI	Time	Time × group	Group
Peppermint	12.60 ± 2.57	11.79, 13.41	9.37 ± 3.78	8.19, 10.56	<0.0001	<0.0001	0.375
Lavender	12.77 ± 2.85	11.88, 13.67	9.50 ± 3.28	8.47, 10.53			
Control	12.50 ± 2.72	11.65, 13.35	11.17 ± 3.32	10.13, 12.21			

^*∗*^Based on repeated measure ANOVA.

## Data Availability

The identified datasets analyzed during the current study are available from the corresponding author upon reasonable request.
